# Undifferentiated pleomorphic sarcoma of the breast with neoplastic fever: case report and genomic characterization

**DOI:** 10.1007/s00432-022-04000-6

**Published:** 2022-05-03

**Authors:** Thilo Gambichler, Kai Horny, Thomas Mentzel, Ingo Stricker, Andrea Tannapfel, Christina H. Scheel, Bertold Behle, Daniel R. Quast, Yi-Pei Lee, Markus Stücker, Laura Susok, Jürgen C. Becker

**Affiliations:** 1 Skin Cancer Center, Department of Dermatology, Ruhr-University Bochum, Bochum, Germany; 2 Department of Dermatology, Christian Hospital Unna, Unna, Germany; 3 Translational Skin Cancer Research, DKTK Partner Site Essen/Düsseldorf, West German Cancer Center, Dermatology, University Duisburg-Essen, Essen, Germany; 4 German Cancer Research Center (DKFZ), Heidelberg, Germany; 5 MVZ Dermatopathology, Friedrichshafen, Germany; 6 Institute of Pathology, Ruhr-University Bochum, Bochum, Germany; 7 Diabetes Division, Department of Internal Medicine, Ruhr-University Bochum, Bochum, Germany

**Keywords:** Undifferentiated pleomorphic sarcoma, High grade sarcoma, Neoplastic fever, Breast cancer

## Abstract

**Purpose:**

Primary breast sarcomas are extraordinary rare, in particular undifferentiated pleomorphic sarcoma (UPS). UPS with neoplastic fever (UPS-NF) of the breast has not been reported yet. Here, we present an extended UPS-NF of the breast including its comprehensive molecular workup.

**Methods:**

A 58-year-old female presented with general malaise, fever spikes, weight loss, and a massively swollen left breast. C-reactive protein and blood leucocytes were significantly increased. However, repeated blood cultures and smears were all sterile. Histopathology of the abscess-forming tumor revealed an undifferentiated malignancy with numerous of tumor giant cells as well as spindle-shaped cells with nuclear pleomorphism and hyperchromasia. Immunohistochemistry demonstrated partial, patchy desmin staining and weak heterogonous neuron-specific enolase immunoreactivity of tumor cells, but a focal staining for Melan-A.

**Results:**

Neither common melanoma driver mutations nor an ultraviolet mutational signature was detected by whole genome sequencing. Using FISH and RT-PCR we also excluded translocations characteristic for clear cell sarcoma. Thus, the diagnosis of inflammatory UPS-NF of the breast was considered highly probable. Despite a complete mastectomy, the tumor recurred after only three months. This recurrence was treated with a combination of ipilimumab and nivolumab based on the primary tumor’s TPS score for PD-L1 of 30%. After an initial response, however, the tumor was progressive again.

**Conclusion:**

We describe here the first case of UPS-NF of the breast, which shows great clinical and histopathologic resemblances to previously reported UPS-NF of other anatomic localizations.

**Supplementary Information:**

The online version contains supplementary material available at 10.1007/s00432-022-04000-6.

## Introduction

Primary sarcomas of the breast are extraordinary rare with < 1% of all breast cancer cases, typically affecting patients aged 55–59 years. Sarcomas of the breast can be classified into histological subtypes as follows: fibroblastic sarcomas, liposarcomas, fibrosarcoma, pleomorphic sarcoma, leiomyosarcoma, rhabdomyosarcoma, and angiosarcoma. Clinically, patients usually present with an unilateral, rapidly growing breast mass, often accompanied by thin vulnerable breast skin and nipple discharge/necrosis (Wang et al. 2011, 2021; Sang et al. 2021; Komaei et al. 2019; Oh et al. 2004; Hashimoto et al. 2021; Kazama et al. 2019; Sajko et al. 2019). Undifferentiated pleomorphic sarcoma (UPS), formerly malignant fibrous histiocytoma, is a high-grade neoplasm, accounting for less than 5% of all adult sarcomas (Wang et al. 2011).

UPS with fever of unknown origin (also known as neoplastic fever) is a specific subtype of UPS (Wang et al. 2011). UPS with neoplastic fever has some clinical features which differentiates it from other UPS subtypes. This UPS subtype can develop anywhere in the body, and its incidence rate is even lower than the ‘normal’ subtypes (Wang et al. 2011, 2021; Sang et al. 2021; Komaei et al. 2019; Oh et al. 2004; Hashimoto et al. 2021; Kazama et al. 2019; Sajko et al. 2019). However, UPS with neoplastic fever (UPS-NF) of the breast has not been reported yet. Here, we present the first case of UPS-NF of the breast which, in addition, we have analysed by whole-exome sequencing (WES).

## Case report

### Clinical presentation

**Fig. 1:**
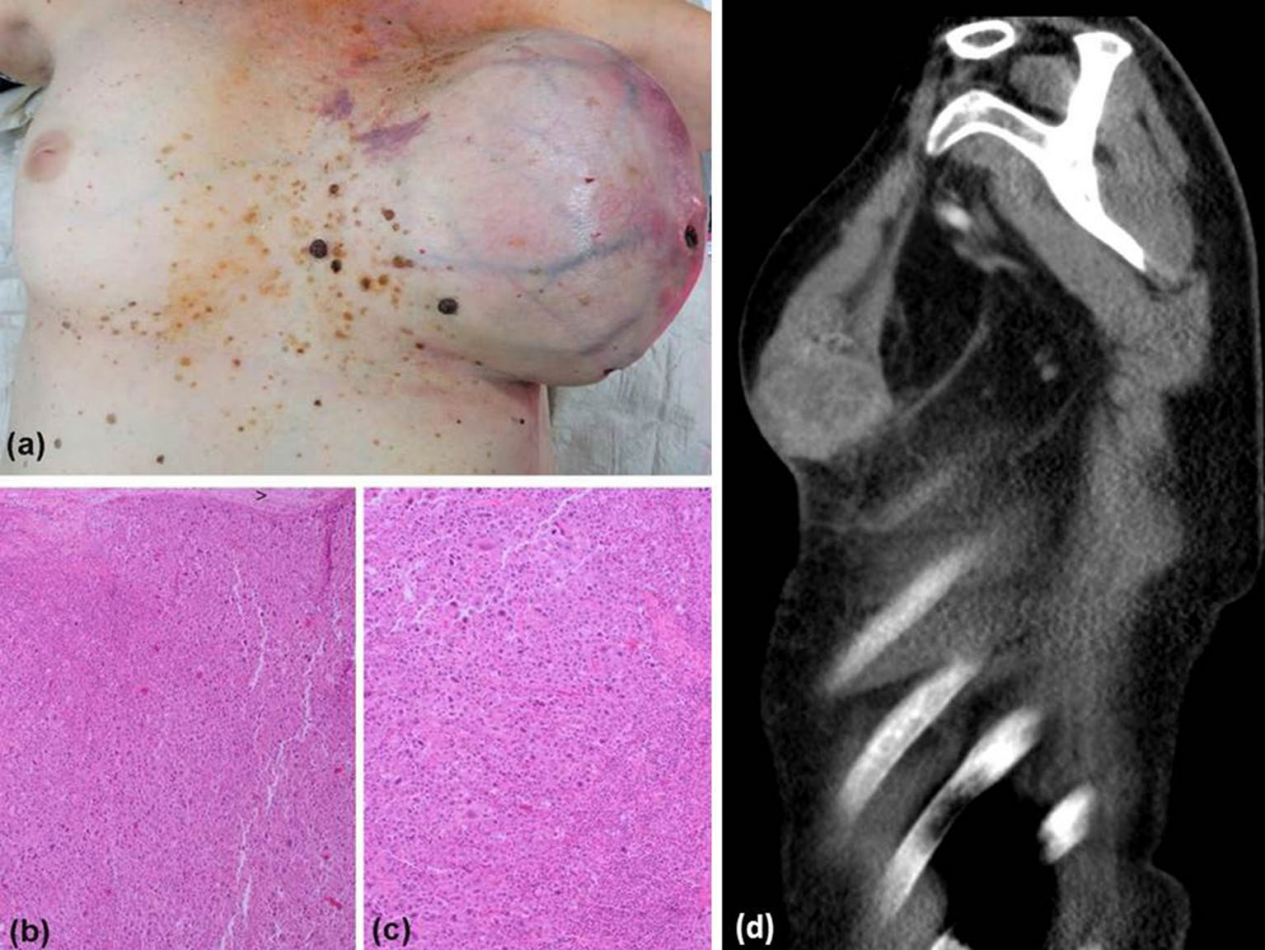
Showing a massively swollen left breast with violaceous erythema, venectases, shiny thinned skin, and necrosis of the nipple in a patient with undifferentiated pleomorphic sarcoma accompanied by neoplastic fever (UPS-NF, **a**). Histologic examination of the completely excised breast tumor revealed a high-grade sarcoma composed of numerous tumor giant cells and atypical spindle-shaped tumor cells (× 40, **b**; × 100, **c**). Computed tomography image demonstrating a huge, partly necrotic tumor of the left breast in a patient with UPS-NF (**d**)

A 58-year-old woman presented with a 3-month history of progressive general malaise, fever spikes, weight loss, and increasing enlargement and discoloration of her left breast. One month earlier, she had noticed an asymptomatic lump deep in her left breast. On examination, there was a massively swollen left breast with violaceous flame-like erythema, shiny thinned skin, and necrosis of the nipple (Fig. 1a, d). C-reactive protein (CRP) was highly elevated with 236 mg/l (< 5). Leucocytes were increased with 16.7/nl (3.9–10.4). As sepsis due to a monstrous breast abscess was suspected, she was urgently admitted to the intensive care unit. However, repeated blood cultures and swabs were all sterile. Incisional biopsy revealed a strong discharge of necrotic tissue and pus-like fluid. Histology of a punch biopsy of this giant abscess-forming tumor revealed a malignancy with spindle-shaped cells, giant cells, and an abundance of necrosis. Provisionally, the diagnosis of a high-grade undifferentiated large-cell malignant neoplasm was made.

Tumor markers in the blood, including S100B, CEA, CA-19–9, CA125, CA15-3, CA72-4, cyfra, synaptophysin, and chromogranin A, were not elevated. Only neuron-specific enolase (NSE) was slightly elevated with 20 µg/l (< 16.3). Initial broad systemic antibiotic therapy did not result in improvement of clinical symptoms and laboratory parameters. However, a few days after complete ablation of the left breast the patient’s condition improved dramatically and was accompanied by a prompt decrease in CRP levels to 10 mg/l.

### Histopathology

Histologic examination of the completely excised breast tumor revealed a high-grade sarcoma composed of numerous tumor giant cells and atypical spindle-shaped tumor cells with prominent nuclear pleomorphism (Fig. 1b, c). In some areas, the mitotic count based on PPH3-staining exceeded over 45 mitoses/mm^2^. Moreover, expansive areas of necrosis were observed. Initially, based on tumor morphology a variety of differential diagnoses were considered, i.e., clear cell sarcoma, pleomorphic rhabdomyosarcoma, metaplastic carcinoma, malignant phyllodes tumor, primary large-cell neuroendocrine carcinoma, and malignant melanoma of the breast. Consequently, immunohistochemistry (IHC) and WES was performed to differentiate these possibilities.

### Immunohistochemistry

IHC was performed for the following parameters, all of which were negative: CEA, EMA, smooth-muscle actin, CK7, CK5/6, CK20, TTF1, MNF116, BerEP4, S100B, SOX10, HMB45, CD10, CD99, CD56, synaptophysin, vimentin, CD68, FLI1, and the hormone receptors ER/PgR/Her2. In addition, focal staining for Melan-A, weak chromogranin A staining, partial, patchy desmin staining and weak heterogonous NSE immunoreactivity was detected in the tumor cells. Ki-67 immunoreactivity was very high with 30% to 90% positively stained tumor cells. The TPS score for PD-L1 was 30%. CD8-positivity was 45% surround the tumor and 7.5% within the tumor. Thus, IHC provided only weak hints towards the identity of the patient’s tumor, with primary melanoma of the breast or undifferentiated sarcoma the most likely differential diagnoses.

### WES and fluorescent in-situ hybridization (FISH)

**Fig. 2:**
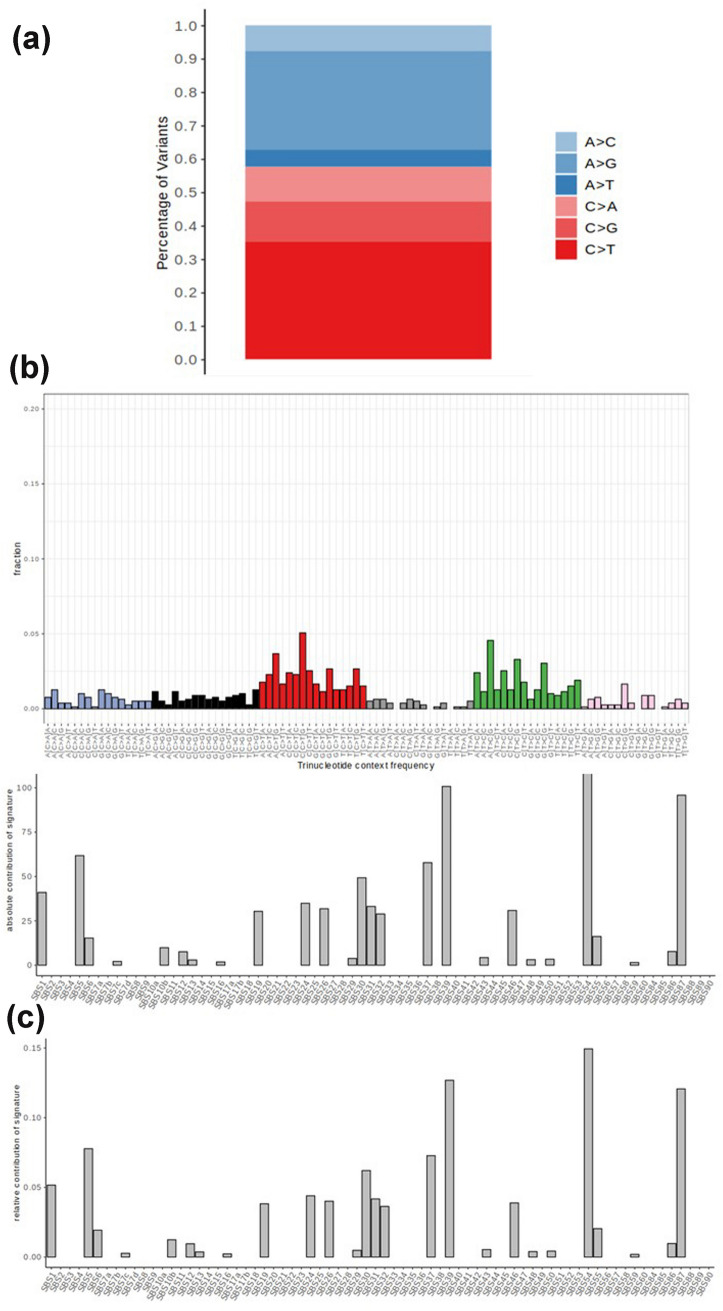
Mutational analysis shows medium tumor mutational burden and absence of UV-associated characteristics. (**a**) Variants per megabasepair (Mbp) colored by nonsynonymous (red shades) and synonymous mutations. (**b**) Fraction of variants colored by transitions and transversions. (**c**) Fraction of variants by transition and transversions broken down by trinucleotide context (*x* axis) and colors by mutation type. Relative contribution of single base substitution (SBS) signatures after fitting trinucleotide context frequency to reference mutational signatures from COSMIC v3.1 database

To obtain insights into the genomic aberrations characterizing this undifferentiated tumor, WES and consequent bioinformatic analysis were performed as previously described (Horny et al. 2021). WES data are illustrated in Fig. 2 and Tab. 1suppl. Because of the observed Melan-A expression in IHC we researched for melanoma-specific genomic aberrations as high tumor mutational burden (TMB), UV-associated mutational signature and characteristic melanoma driver mutations. Since we have not sequenced a normal control, variants were filtered for SNPs by comparing variant allele frequency with the *gnomAD* genome database. With respect to TMB, we identified 952 variants (19.17 mutations per mega base pair) and no over-representation of C > T mutations indicating absence of UV-associated mutational signature (Fig. 2a). We further analysed the trinucleotide context frequency and fitted them to COSMIC v3.1 single base substitution (SBS) reference signatures (Fig. 2b). Both clearly show the absence of UV-associated mutational signatures (SBS7a-d). Common melanoma driver mutations in *BRAF, NRAS, KIT, RB1* genes, were not detected, but one missense mutation in TP53 (dbSNP: rs876660825).

Since genomic data were not suggestive of melanoma or any other specific entity, we focused our attention on sarcoma-specific genetic aberrations, particularly since clear cell sarcoma of soft tissue are often Melan-A positive, as well. To this end, we queried the presence of inter-chromosomal translocations *EWSR1-ATF1* and *EWSR1-CREB1* which are usually present within clear cell sarcoma of soft tissue. We checked the presence of chimerics reads and inter-chromosomal read-pairs in *EWSR1, ATF1* and *CREB1* with the integrative genome viewer (IGV) and could not find evidence for translocations. Further, these translocations were not detected using FISH and RT-PCR in several tumor specimens of the primary tumor and cutaneous relapses. Taken together, we can also exclude a malignant melanoma and clear cell sarcoma in the diagnosis.

### Diagnosis, treatment and follow-up

**Fig. 3:**
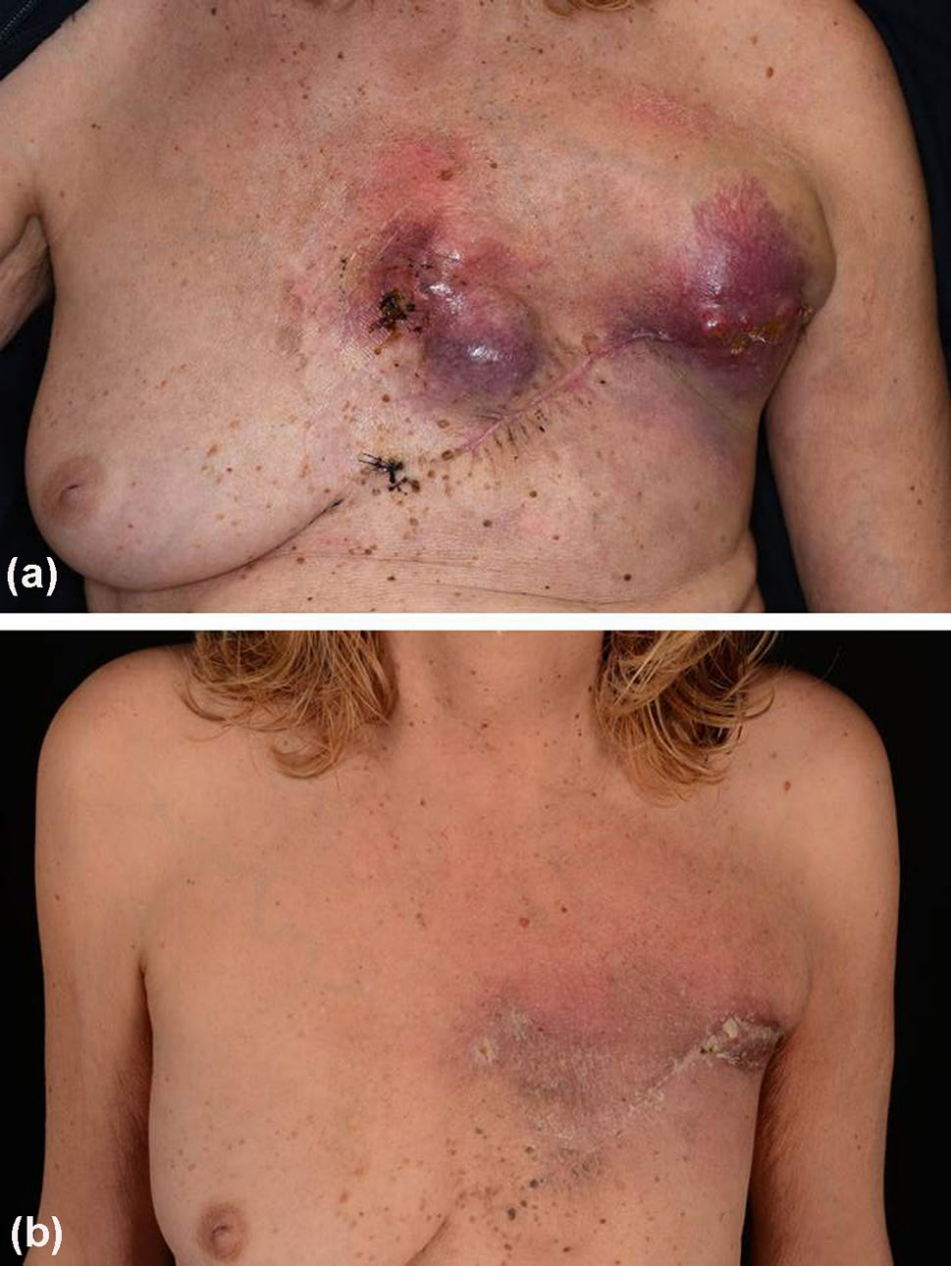
Showing a patient with a massive relapse of an undifferentiated pleomorphic sarcoma of the breast (**a**). Three cycles of combined immunotherapy and radiotherapy resulted in significant regression of the tumor masses (**b**)

Eventually, a diagnosis of inflammatory UPS-NF was deemed most likely, given the lack of any indicative immunohistological or genomic findings suggesting other entities together with clinical characteristics. With respect to treatment, complete mastectomy was followed with staging diagnostics including lymph node ultrasound, thoracic and abdominal computed tomography (CT) as well as cranial magnetic resonance imaging. On this basis, the patient was declared tumor-free and no further treatment was planned. However, only three months after initial breast ablation, multiple tumor nodules recurred on the left thoracic wall and axillar region (Fig. 3a). Lactate dehydrogenase (LDH) was elevated at 370 U/l (135–214), but other tumor markers such as serum S100B remained within the normal range. CT scans confirmed pectoral, thoracic, and axillar tumor masses on the patient’s left side. Moreover, a new suspect lesion was detected in the upper lobe of the right lung. Since strong PD-L1 expression was detected in the primary tumor, the interdisciplinary tumor board recommended combined radiotherapy (15 × 2.7 Gy) and immunotherapy using immune checkpoint inhibitors (ICI). Consequently, a combined regimen of ipilimumab (3 mg/kg body weight) and nivolumab (1 mg/kg/body weight) was administered. However, following the first cycle of combined immunotherapy, the patient developed severe ketoacidosis due to autoimmune-induced diabetes mellitus, which required insulin treatment. Combined immunotherapy and radiotherapy were continued and after the third cycle, the skin metastases on the left thoracic side and axillar region had significantly regressed (Fig. 3b). CT scans showed that the previously noted lung lesion in the right upper lobe had not increased in size. However, new mediastinal lymph node metastases were detected. LDH levels were within the normal range with 149 U/l (135-214). On the basis of this mixed response, the patient received a fourth cycle of combined immunotherapy and then continued nivolumab monotherapy every four weeks. Unfortunately, after two cycles of nivolumab monotherapy, skin lesions reappeared on the left chest wall. CT scans revealed that both pulmonal and mediastinal metastases had significantly progressed, although no other organ manifestations. However, there were no other organ manifestations. LDH levels remained in the upper normal range with 204 U/l (135-214). Consequently, immunotherapy was discontinued and instead, chemotherapy planned. However, the patient declined chemotherapy and wished, instead, to seek alternative medicine treatments elsewhere. Three months later, the patients returned to our department with further progressed pulmonal metastases and a new cutaneous tumor mass on the chest. Again, she refused systemic treatment, but instead decided on sole excision of the cutaneous metastasis. Nonetheless, the patient died from her tumor 15 months after diagnosis.

## Discussion

This case was associated with extraordinary diagnostic challenges, since the patient presented with signs of sepsis and clinical findings highly suggestive of a monstrous breast abscess. After biopsy pointed to a malignancy, the lack of specific immunohistology markers or genomic signatures further complicated making an accurate diagnosis. In addition, neoplastic fever, which typically presents with elevated parameters of inflammation without any evidence for an infection, is a relatively rare event, accounting for less than 3% of patients with fever of unknown origin (Sørensen et al. 2005). Wang et al. (2011) reported two patients with highly aggressive, histologically identical tumors presenting as solitary, tender, necrotising, masses located to the bone that were associated with fever, leukocytosis and, similarly to our case, no evidence for infection. Histopathology revealed large monomorphic epithelioid cells with vesicular nuclei and abundant eosinophilic cytoplasm surrounded by numerous neutrophils and eosinophils which formed sterile microabscesses. IHC as well as electron microscopy did not reveal any specific differentiation or tissue origin. In contrast to these findings, the tumor discussed here clearly showed polymorphic cell growth. Moreover, a recent retrospective study provided detailed information on seven UPS-NF patients (three males, four females) (Wang et al. 2021). In all patients, the primary lesions (diameter 4.8–18.0 cm) were located in the extremities and intermuscular space. Of note, neoadjuvant radiotherapy and chemotherapy did not relieve neoplastic fever, however, similar to the case presented here, extensive resection of the primary tumor resulted in prompt dissolution of neoplastic fever (Wang et al. 2011, 2021). Moreover, it was reported that patients experienced at least one postoperative recurrence and fatal pulmonary metastatic disease with six patients still alive three years after surgery (Wang et al. 2011). Apart from this retrospective study, undifferentiated embryonal liver sarcoma in childhood and undifferentiated sarcomas in other anatomic sites have been reported in conjunction with fever and other signs of inflammation (Hashimoto et al. 2021; Kazama et al. 2019; Sajko et al. 2019; Mass and Talmon 2018; Horny et al. 2021; Sørensen et al. 2005; He et al. 2014). In some cases, elevated levels of tumor-released cytokines have been observed as the likely cause of systemic inflammation (Kazama et al. 2019; Yanagisawa et al. 2015; Li et al. 2010). Taken together, we did not find a matching case of inflammatory UPS-NF, but there are cases of soft tissue sarcomas of different subtypes in other locations that presented with signs of systemic inflammation most likely linked to cytokine production of the primary tumor.

Similar to two patients with UPS-NF reported by Wang et al. (2021), we did not observe any characteristic immunoreactivity for any marker investigated. Remarkably, even broadly mesenchymal markers vimentin and CD68 were negative, a finding typically seen in undifferentiated pleomorphic sarcomas. Because of the focal Melan-A expression we initially considered primary melanoma of the breast parenchyma as a differential diagnosis, which is exceptionally rare with only 15 confirmed cases in the literature (Snashall et al. 2020). However, genomic analysis by WES largely excluded melanoma, as no melanoma-specific mutations and mutational signatures were detected. IHC and genomic data carry particular weight with respect to melanoma, since it was shown previously that even highly un- or transdifferentiated melanomas display characteristic markers and mutational profiles (Ferreira et al. 2022; Agaimy et al. 2016). Moreover, during the entire course of her disease the patient did not show abnormal S100B levels.

Indeed, genomic analysis by WES did not shed any light with respect to the subtype of the sarcoma described here. Since Melan-A expression also occurs in the exceedingly rare cases of clear cell sarcoma of the breast (Pollard et al. 1990; Ibrahim et al. 2018), we checked for the frequent translocations *EWSR1-ATF1* and *EWSR1-CREB1*. We could not detect these translocations within the WES data and by FISH analysis. Thus, mostly through exclusion of other possibilities and based on clinical and morphological findings, we determined that the most accurate diagnosis for this case would be that of a primary breast UPS-NF that initially imposed as a massive breast abscess accompanied by systemic inflammation.

Upon recurrence of the primary tumor together with the appearance of a suspect lesion in the lung, the interdisciplinary tumor board recommended double immune checkpoint blockade together with radiotherapy. Indeed, given the lack of any other effective systemic therapies in soft tissue sarcomas and the general interest in immunotherapy, a few studies using ICI and other avenues of immunotherapy have been conducted (Ayodele and Abdul Razak 2020). There are many ongoing phase I/II studies particularly determining the effects of double immunotherapy in conjunction with radiotherapy, as our patient received. Moreover, IHC of the present case revealed strong PD-L1 staining in the primary tumor, lending further support for first-line immunotherapy upon recurrence.

Moreover, WES detected a relatively high TMB, which is emerging as a predictor for response to ICI (Hu et al. 2020). In general, it is also thought that PD-L1 positivity of tumor cells “hot” (CD8 +), inflammatory tumors, as was the case discussed here, respond better to immunotherapy (Ayodele and Abdul Razak 2020). Indeed, the patient appeared to at least achieve a mixed response under double immunotherapy, but unfortunately, progressed three months later. Here, it would have been highly desirable to obtain another tumor sample to assess tumor evolution both using IHC for PD-1 and at the molecular level by another round of WES. More studies aimed at understanding the dynamics of metastatic progression under immunotherapy are, of course, urgently needed, while, at the same time, remain particularly challenging for rare entities such as soft tissue sarcomas. D’Angelo et al. (2018) recently demonstrated that nivolumab alone does not warrant further study in an unselected sarcoma population given the limited efficacy. However, nivolumab combined with ipilimumab demonstrated promising efficacy in certain sarcoma subtypes (e.g., UPS), with a manageable safety profile comparable to current available treatment options. Nakata et al. (2021) also reported that sarcomas with a greater TMB, such as UPS, myxofibrosarcoma and dedifferentiated liposarcomas, may be good candidates for ICI. Another avenue to boost response to immunotherapy might lie in identifying rational combinations of immune- and targeted therapy. For example, amplification of genes coding for histone deacetylases was recently reported to occur in about 20% of soft tissue sarcoma samples (Que et al. 2021). Consequently, combination treatment with a PD-1 and HDAC inhibitor proved effective in a preclinical model. However, for a patient as presented here, with no targetable genomic aberrations, achieving durable responses will remain to pose extraordinary challenges.

In conclusion, we described the first case of UPS-NF of the breast, which clinically and histopathologically resembled previously reported UPS-NF of other anatomic sites.

## Supplementary Information

Below is the link to the electronic supplementary material.

Supplementary file1 (CSV 1199 kb)
